# Co-delivery of Cas9 mRNA and guide RNAs for editing of LGMN gene represses breast cancer cell metastasis

**DOI:** 10.1038/s41598-024-58765-6

**Published:** 2024-04-06

**Authors:** Yue Wang, Yatu Peng, Guanghui Zi, Jin Chen, Baowei Peng

**Affiliations:** 1 College of Pharmacy, Dali University, 2 HongShen Road, Dali, 671003 Yunnan China; 2 JinCai High School, 2788 Yang Gao Middle Road, Pudong New District, Shanghai, 200135 China

**Keywords:** Cancer, Cell biology

## Abstract

Legumain (or asparagine endopeptidase/AEP) is a lysosomal cysteine endopeptidase associated with increased invasive and migratory behavior in a variety of cancers. In this study, co-delivery of Cas9 mRNA and guide RNA (gRNA) by lipid nanoparticles (LNP) for editing of LGMN gene was performed. For in-vitro transcription (IVT) of gRNA, two templates were designed: linearized pUC57-T7-gRNA and T7-gRNA oligos, and the effectiveness of gRNA was verified in multiple ways. Cas9 plasmid was modified and optimized for IVT of Cas9 mRNA. The effects of LGMN gene editing on lysosomal/autophagic function and cancer cell metastasis were investigated. Co-delivery of Cas9 mRNA and gRNA resulted in impaired lysosomal/autophagic degradation, clone formation, migration, and invasion capacity of cancer cells in-vitro. Experimental lung metastasis experiment indicates co-delivery of Cas9 mRNA and gRNA by LNP reduced the migration and invasion capacity of cancer cells in-vivo. These results indicate that co-delivery of Cas9 mRNA and gRNA can enhance the efficiency of CRISPR/Cas9-mediated gene editing in-vitro and in-vivo, and suggest that Cas9 mRNA and gRNA gene editing of LGMN may be a potential treatment for breast tumor metastasis.

## Introduction

Legumain was originally discovered in common legumes^1,2^. In 1996, it was first identified as a specific protease encoded by the LGMN gene on chromosome 14 in humans^3^. Legumain selectively cleaves substrates after asparagine (N) residues^4^, thus earning the name asparagine endopeptidase (AEP)^1^. AEP has multiple functions, including in maturation of other proteases such as MMPs and in immune regulation^5,6^. It has also been shown to participate in the regulation of vascular homeostasis, which plays a significant role in both cardiovascular and cerebrovascular diseases^7^. Recently, researchers have found that legumain is present in a variety of solid tumors, such as breast cancer^8,9^ and melanoma^10^. In addition, it is also associated with increased aggressiveness and migratory behavior in cancers, including breast, prostate, colorectal, and gastric cancers^11–13^. Not surprisingly, legumain has been associated with poor prognosis in several types of malignant tumors^11,14^.

The CRISPR-Cas system is an innate immune system in prokaryotes that degrades invading foreign DNA^15^. In recent years, the CRISPR-Cas9 genome editing technology has emerged as a potentially powerful tool for treating human diseases^16,17^. As a tool of gene therapy, it can treat various diseases through DNA editing technology^18^. For many solid tumors, such as breast cancer, the metastasis and spread of tumor cells are important causes of death in cancer patients and also major challenges in cancer treatment^19^. The use of CRISPR-Cas9 gene editing technology to knock out pathogenic or related target genes in the genome to treat cancer is of great significance for tumor gene therapy^20,21^. Recent studies have found that AEP is up-regulated in lysosomes in cancer cell^22^. Since lysosomes act as cellular centers for signaling such as Akt activation^23–25^, which makes AEP an attractive therapeutic target for the treatment of tumor metastasis. Knocking out AEP through CRISPR-Cas9 gene editing technology to inhibit tumor metastasis may be a feasible, effective anti-tumor strategy.

When considering gene-editing strategies as a therapeutic approach for cancer treatment, it's critical to anticipate and understand potential resistance mechanisms that cancer cells might develop. This is important for improving the effectiveness of the treatment and for the development of strategies to overcome resistance^26,27^. Some potential resistance mechanisms include the following: (1) Cancer cells could develop mutation in the target gene that prevent the gene-editing tool from recognizing or binding to its intended sequence, thus rendering the therapy ineffective^28^. (2) Non-homologous end joining (NHEJ) is critical for the repair of double strand break induced by Cas9 and lead to the introduction of insertions or deletions (indels) at the site of gene editing, it can also result in unintended mutations that confer resistance^28–30^. (3) Cancer cells may develop resistance through epigenetic modifications that affect gene expression without altering the DNA sequence, potentially reducing the efficacy of gene-editing strategies^28^.

Gene editing technologies, particularly CRISPR-Cas9 and its successors, have revolutionized the field of genetics^31^, providing unprecedented precision in modifying DNA^32^. While the therapeutic potential of gene editing is immense^33^, it raises several socio-ethical concerns that must be carefully considered, especially when it comes to human treatment. Here are some key implications: (1) One of the primary ethical concerns is the safety of gene editing in human beings. Ensuring that gene editing is safe and predictable is crucial before it can be widely adopted^34^. (2) Germline editing produces changes in the genome would be passed on to future generations. This has profound implications for the human gene pool and raises questions about the rights of future individuals^34^. (3) different cultures and religions have varying views on the acceptability of intervening in the natural genetic order^35^. Some may see gene editing as playing God or as an unnatural act, which could lead to significant public debate and conflict.

In this study, we designed legumain specific gRNA pairs, created novel ways of construction in-vitro transcription templates for gRNA expression, and verified the effectiveness of the gRNAs. Meanwhile, we also constructed a Cas9 in-vitro transcription template. The LGMN gene was edited by co-delivery of Cas9 mRNA and gRNAs in breast cancer cells in-vitro and in-vivo, and assayed AEP expression, enzymatic activity, lysosomal function, autophagy, colony formation, and cancer cell metastasis. Knockout of AEP in breast cancer cells inhibited their migration and invasion both in-vitro and in-vivo. Our results indicate knocking down lysosomal protease to suppresses tumor metastasis by co-delivery Cas9 mRNA and gRNAs is feasible.

## Results

### Construction of gRNA expression plasmid and IVT template

**Figure 1:**
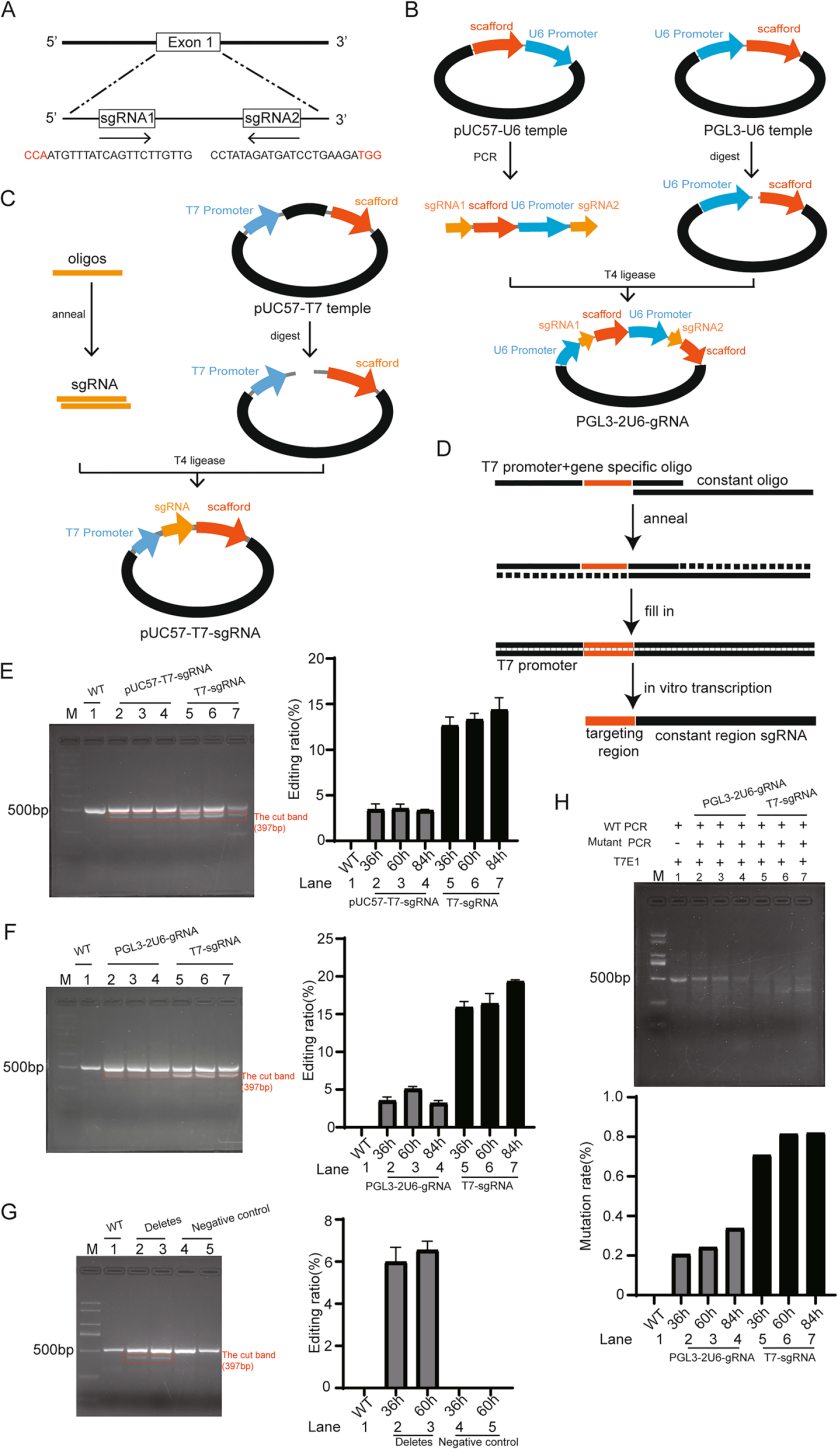
Construction of gRNA templates and comparison of efficacy of gRNAs derived from different templates. (**A**) A pair of gRNAs was designed to induce cleavage in exon 1 of the human LGMN gene by Cas9. (**B**) Construction of plasmid containing dual U6 promoter-driven gRNAs. (**C**) Construction of plasmid template for IVT of gRNA by ligation of annealed gRNA oligos with digested pUC57-T7. (**D**) Construction of T7-gRNA oligo template. (**E**) Comparison of gene-editing efficiency of gRNAs derived from different templates at 36 h, 48 h, and 84 h after transfection of cells with gRNAs and Cas9 plasmids by Lipofectamine 3000. Left: Target gene was amplified by PCR. Right: The gray value of the band was calculated to indicate the editing ratio, data are mean ± SEM of triplicates from one experiment. (**F**) Comparison of gene editing efficiency of pGL3-2U6-gRNA and gRNAs derived from T7-gRNA oligo template in the presence of Cas9 plasmids at 36 h, 48 h, and 84 h after transfection by PCR. Right: The gray value of the band was calculated to indicate the editing ratio, data are mean ± SEM of triplicates from one experiment. (**G**) The editing efficiency of gRNA was calculated, and the non-targeting control gRNA was used as the negative control. Right: The gray value of the band was calculated to indicate the editing ratio, data are mean ± SEM of triplicates from one experiment. (**H**) Gene editing validation by T7E1 endonuclease assay. Right: The gray value of the band was calculated to indicate the mutation rate. Original gels are presented in Supplementary Fig. 1. The error bars represent the SEM.

Legumain (or AEP) specific gRNA sequences, 20 bases 5′ upstream of PAM, were selected manually (Fig. 1A). To construct the dual gRNAs expressing plasmid, gRNA scaffold-U6 promoter was amplified from pUC57-U6-template by primers containing the pair of gRNAs (Fig. 1B). The amplified gRNA scaffold-U6 promoter sequence was then inserted between the U6 promoter and the gRNA scaffold in the backbone plasmid pGL3-U6 by restriction enzyme cloning to obtain pGL3-2U6-gRNA, so that each gRNA would be driven by a U6 promoter and containing a gRNA scaffold, i.e., U6-sgRNA1-scaffold-U6-sgRNA2-scaffold (Fig. 1B).

Two templates for IVT of gRNA were also constructed. To construct plasmid gRNA template, annealed gRNA oligos were inserted between the T7 promoter and the gRNA scaffold in pUC57-U6-template by restriction enzyme cloning using BsaI enzyme to obtain pUC57-T7-gRNA (Fig. 1C). To construct universal linear DNA template for IVT of gRNA, a gene specific oligo containing gRNA downstream of T7 promoter was partially annealed to a constant oligo containing gRNA scaffold (Fig. 1D). After fill-in to become double-stranded DNA, PCR reaction was used to amplified the entire sequence, dubbed as T7-gRNA, and purified DNA was used as gRNA IVT template.

### Comparison of gene-editing efficiency of gRNAs derived from different IVT templates

Gene-editing efficacy of gRNA pairs were determined by co-transfection of cells with gRNAs encoding and Cas9 encoding plasmids (data not shown). The optimal gRNA pair was selected for further study. To evaluate gene-editing efficiency of gRNAs derived from different IVT templates, MDA-MB-231 cells were co-transfected with gRNAs and plasmids encoding Cas9. Genomic DNA encompassing the intended deletion region was amplified by PCR with specific primers, PCR products were resolved by electrophoresis on 2% agarose gel, and deletion ratio was calculated. As shown in Fig. 1E, gRNAs derived from T7-gRNA were far more efficient in mediating gene-editing by Cas9 than that derived from linearized pUC57-T7-gRNA.

Next, gene-editing efficacy of gRNAs derived from T7-gRNA was compared directly to the pGL3-2U6-gRNA plasmids. We found that gRNAs derived the T7-gRNA template exhibited higher efficiency than pGL3-2U6-gRNA plasmid in mediating gene-editing by Cas9 (Fig. 1F), as shown by deletion of genomic DNA in PCR products. Next, we evaluated the editing efficiency of gRNAs side-by-side with non-targeting control gRNAs (Fig. 1G). In addition, we assessed the deletion/insertion in target gene by using T7E1 assay (Fig. 1H). Clearly, T7E1 mediated more severe cleavage of target DNA derived from cells transfected with gRNAs and plasmids encoding Cas9 than target DNA derived from cells treated with pGL3-2U6-gRNA plasmids and Cas9 plasmids, indicating more efficient gene-editing mediated by the former.

### Transform of Cas9 plasmid, IVT, and validation of Cas9 mRNA in gene-editing

**Figure 2:**
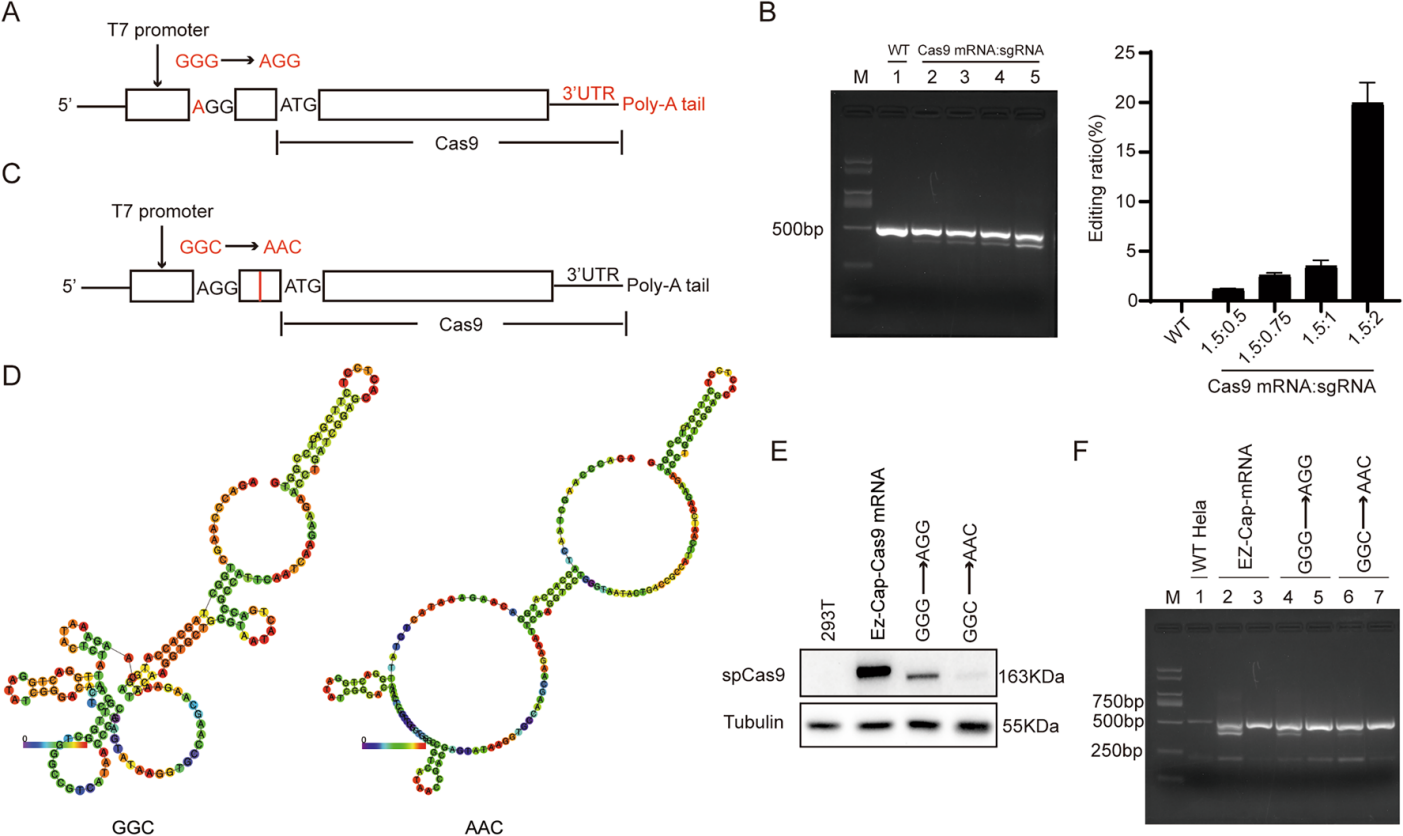
Modification Cas9 plasmid and validation of the Cas9 mRNA in gene-editing. (**A**) Schematic diagram of modified Cas9 plasmid for IVT with point mutation to facilitate co-transcriptional capping, 3′UTR and PolyA tail added. (**B**) Validation of Cas9 mRNA in gene-editing in the presence of incremental amounts of gRNAs. Cas9 mRNA and gRNA at mass ratios of 1.5:0.5, 1.5:0.75, 1.5:1, and 1.5:2 were investigated. Gels shown are from one experiment representative of three. Data are mean ± SEM of triplicates from one experiment. The error bars represent the SEM. (**C**) Schematic diagram of further modification of Cas9 plasmid for IVT with GGC to AAC mutation in the untranslated region. (**D**) Predicted minimum energy structure of RNA folding with and without the GGC to AAC point mutation. The 5′UTR plus the immediate 168 bps in the Cas9 open reading frame was examined for RNA folding. (**E**) Cas9 protein expression after transfection of Cas9 mRNAs into 293 T cells. EZ-Cap-Cas9 mRNA as a positive control. (**F**) The efficiency of gene-editing of Cas9 mRNAs in the presence of gRNAs was evaluated in Hela cells. Original blots/gels are presented in Supplementary Fig. 1.

To obtain Cas9 mRNA IVT template, we used pST1374-N-NLS-flag-link-Cas9 as start material which contains a bacterial promoter T7 promoter located between the eukaryotic promoter CMV and the start codon. Fist, we added a 3′ UTR and a polyA tail to the end of the Cas9 open reading frame by seamless cloning (Fig. 2A). To facilitate co-transcriptional capping of mRNA, the trinucleotide GGG in the plasmid following the T7 promoter was mutated to AGG through a point mutation (Fig. 2A). The modified Cas9 plasmid (GGG to AGG mutation) were linearized and used as template for in-vitro transcription with co-transcriptional capping. We investigated the gene-editing efficacy of resulting Cas9 mRNA in MDA-MB-231 cells with different ratio of Cas9 mRNA to gRNA. Gene-editing efficiency was examined by amplification of target gene by PCR. The results indicated that the Cas9 mRNA was effective in gene editing (Fig. 2B). At equal amount of Cas9 mRNA, the more the gRNAs used, the higher the gene editing efficiency (Fig. 2B).

There is a 22-bp 5′UTR sequence, i.e., AGACCCAAGCTGGCTAGCACC, between the T7 promoter and start codon in the plasmid. We hypothesized this untranslated sequence would participate in the folding of the final RNA. We used RNA fold, a thermodynamics-based RNA secondary structure folding algorithm^36^, to evaluate folding tendency of this untranslated sequence in conjunction with the first 168 bp of the Cas9 open reading frame. According to RNA fold, the trinucleotide GGC in the untranslated sequence play an important role in RNA folding (Fig. 2C). We mutated the trinucleotide GGC to AAC by point mutation. This modification in the non-coding region after the T7 promoter is predicted to reduce RNA stability by increasing minimum free energy from − 36.60 to − 28.30 kcal/mol (Fig. 2D).

The Cas9 mRNAs derived from the AGG IVT template and from the AAC IVT template (GGC to AAC mutation) were transfected into 293 T cells. Commercial Cas9 mRNA was used as control. Western blot results showed that Cas9 mRNA derived from the AGG IVT template produced Cas9 protein as expected (Fig. 2E), although at lower levels than the commercial Cas9 mRNA, indicating the template or IVT procedure needed further optimization. On the other hand, Cas9 mRNA derived from the AAC IVT template (GGC to AAC mutation) almost did not produce Cas9 protein (Fig. 2E), consistent with compromised RNA folding upon GGC to AAC mutation predicted by RNA fold (Fig. 2C). In-vitro gene-editing experiment confirmed that Cas9 mRNA derived from mutant template was less effective in gene-editing (Fig. 2F).

### AEP knockdown by co-delivered Cas9 mRNA/gRNAs compromised autophagic and lysosomal degradation

**Figure 3:**
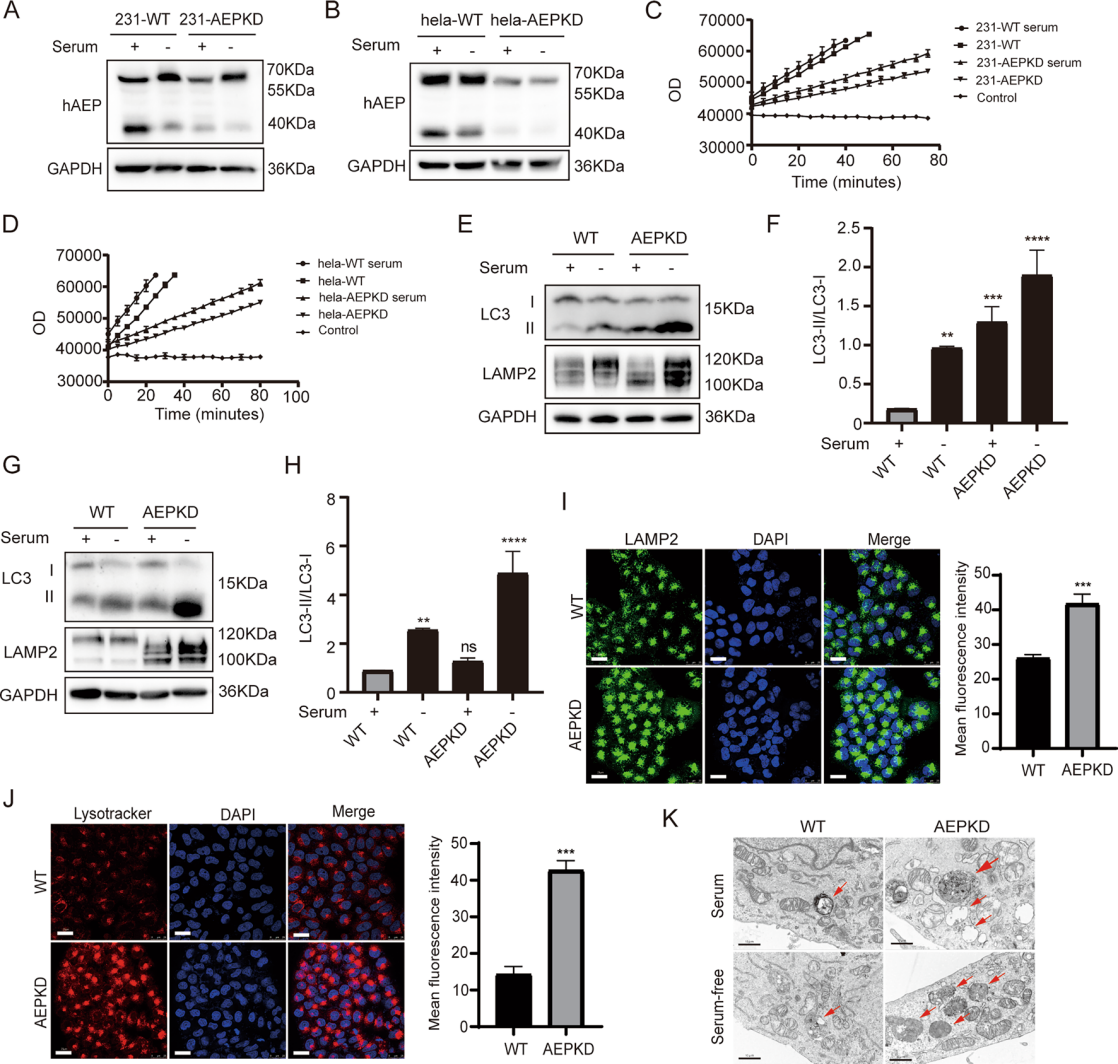
AEP knockdown by co-delivered Cas9 mRNA and gRNAs reduced autophagic and lysosomal degradation. (**A**,**B**) Expression of AEP protein was analyzed by Western blotting in MDA-MB-231 (**A**) and HeLa cells (**B**) after gene-editing (AEPKD) under serum repletion or serum depletion. (**C**,**D**) AEP enzymatic activity was measured by substrate Z-Ala-Ala-Asn-AMC cleavage assay in lysates of MDA-MB-231 (**C**) and Hela cells (**D**) after gene-editing (AEPKD). (**E**,**F**) LC3 proteins were analyzed by Western blotting in MDA-MB-231 cells. Representative images of blots are shown (**E**), and the ratio of LC3-II/LC3-I was quantified (**F**), data are mean ± SEM of three independent experiments; statistics are unpaired t-test. (**G**,**H**) LC3 proteins were analyzed by Western blotting in Hela cells. Blots shown are from one experiment representative of three (**G**), and the ratio of LC3-II/LC3-I was quantified (**H**), data are mean ± SEM of three independent experiments; statistics are unpaired t-test.. (**I**) Confocal images of WT and AEP gene-edited HeLa cells immunostained with antibodies against LAMP2. Images are from one experiment representative of three, statistics are unpaired t-test. Scale bar: 25 μm. (**J**) Confocal images of WT and AEP gene-edited HeLa cells labeled with lysosome probe Lysotracker. Images are from one experiment representative of three, statistics are unpaired t-test. Scale bar: 25 μm. (**K**) Transmission Electron Microscopes examination of autophagosomes and autolysosomes in WT and AEP gene-edited MDA-MB-231 cells. Arrows indicate autophagosomes and autolysosomes with undegraded contents. Scale bar: 10 μm. The error bars represent the SEM. Compared with the wild-type group. **P < 0.005, ***P < 0.001, ****P < 0.0001. Original blots are presented in Supplementary Fig. 1.

Cas9 mRNA and gRNAs co-delivered by Lipofectamine 3000 reduced the expression of AEP protein in both MDA-MB-231-cells (Fig. 3A) and Hela cells (Fig. 3B) (hereafter AEPKD cells), consistent with LGMN gene cleavage by Cas9 mRNA and gRNAs (Fig. 2B).

In contrast, CRISPR/Cas9 gene-editing reduced both pro-AEP and active-AEP (Fig. 3A,B). Substrate Z-Ala-Ala-Asn-AMC cleavage assay demonstrated AEP activity was reduced in cells after LGMN gene-editing (Fig. 3C,D). Since AEP is lysosomal protease, we examined the levels of lysosomal/autophagic protein expression in MDA-MB-231 cells after LGMN gene editing by western blotting (Fig. 3E). The results showed accumulation of LC3-II protein, increase in LC3-II/LC3-I ratio, and accumulation of lysosomal associated membrane protein LAMP2 in cells underwent LGMN gene-editing (AEPKD cells) (Fig. 3F), indicating compromised autophagic/lysosomal degradation. Similar results were observed in LGMN gene-edited HeLa cells (Fig. 3G,H).

Next, we further explored lysosomal homeostasis by LAMP2 immunostaining (Fig. 3I) and Lysotracker labeling (Fig. 3J). We found that both the LAMP2 staining and Lysotracker labeling resulted in significantly higher fluorescence intensity in AEPKD cells than that in WT cells (Fig. 3I,J), indicating accumulation of lysosomes. Moreover, transmission electron microscopy showed an increase in the number of autophagic vesicles, a reduction in autophagic content degradation in AEPKD cells under both serum repletion and serum depletion (Fig. 3K). Together, these results suggest LMGN gene-editing by Cas9 mRNA and gRNAs compromised autophagic and lysosomal degradation in cells.

### AEP knockdown by co-delivered Cas9 mRNA/gRNAs impaired cancer cell survival, migration, and invasion

**Figure 4:**
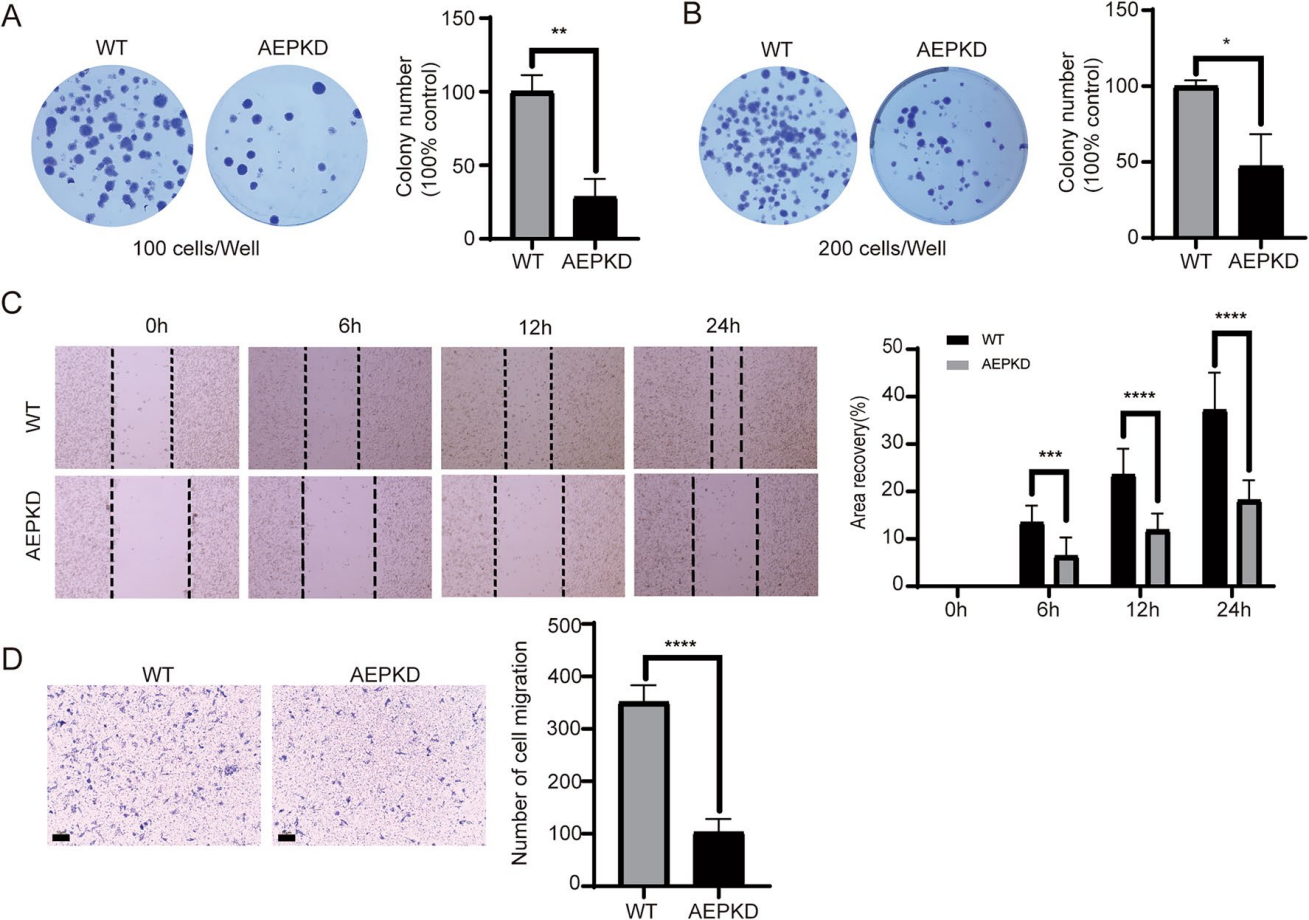
AEP knockdown by co-delivered Cas9 mRNA and gRNAs impaired cancer cell proliferation, migration, and invasion. (**A**,**B**) Survival of WT and AEPKD MDA-MB-231 cells were assessed by colony formation assay. 100 cells (**A**) or 200 cells (**B**) were plated in each well of 6-well plate and cultured for 2-weeks. (**C**) Migration of WT and AEPKD MDA-MB-231 cells was examined in a wound-healing assay. The cell monolayer was scratched with pipette tips, and the scratches were imaged at 0, 6, 12, and 24 h afterwards. (**D**) Invasion ability of WT and AEPKD MDA-MB-231 cells was examined by transwell assay. The cells were fixed and stained with crystal violet. Scale bar: 10 μm. (**D**) The effect of LGMN gene editing on migration was assessed in transwell migration assay. Graphs shown are from one experiment representative of three. The data are presented as mean ± SEM. Statistics are unpaired t-test. **P < 0.005, ***P < 0.001, ****P < 0.0001, compared with the wild-type group.

To investigate the consequence of LGMN gene-editing, we performed colony formation assay, wound healing assay, and transwell assay. We found LMGN gene-editing by co-delivered Cas9 mRNA and gRNAs significantly reduced colony formation capability of MDA-MB-231 cells (Fig. 4A,B), indicating impaired survival of AEPKD cells. In addition, wound healing assay and transwell assay were performed to examine cell migration and invasion. Wound healing assay showed that AEPKD cells migrated slower than wild-type cells (Fig. 4C). And transwell assay showed that AEPKD cells were less invasive than wild-type cells (Fig. 4D). Together, these results indicate that AEP knockdown by Cas9 mRNA and gRNAs impairs cancer cell survival, migration, and invasion.

### Effect of in-vivo LGMN gene-editing by SORT-LNP co-delivered Cas9 mRNA/gRNAs

**Figure 5:**
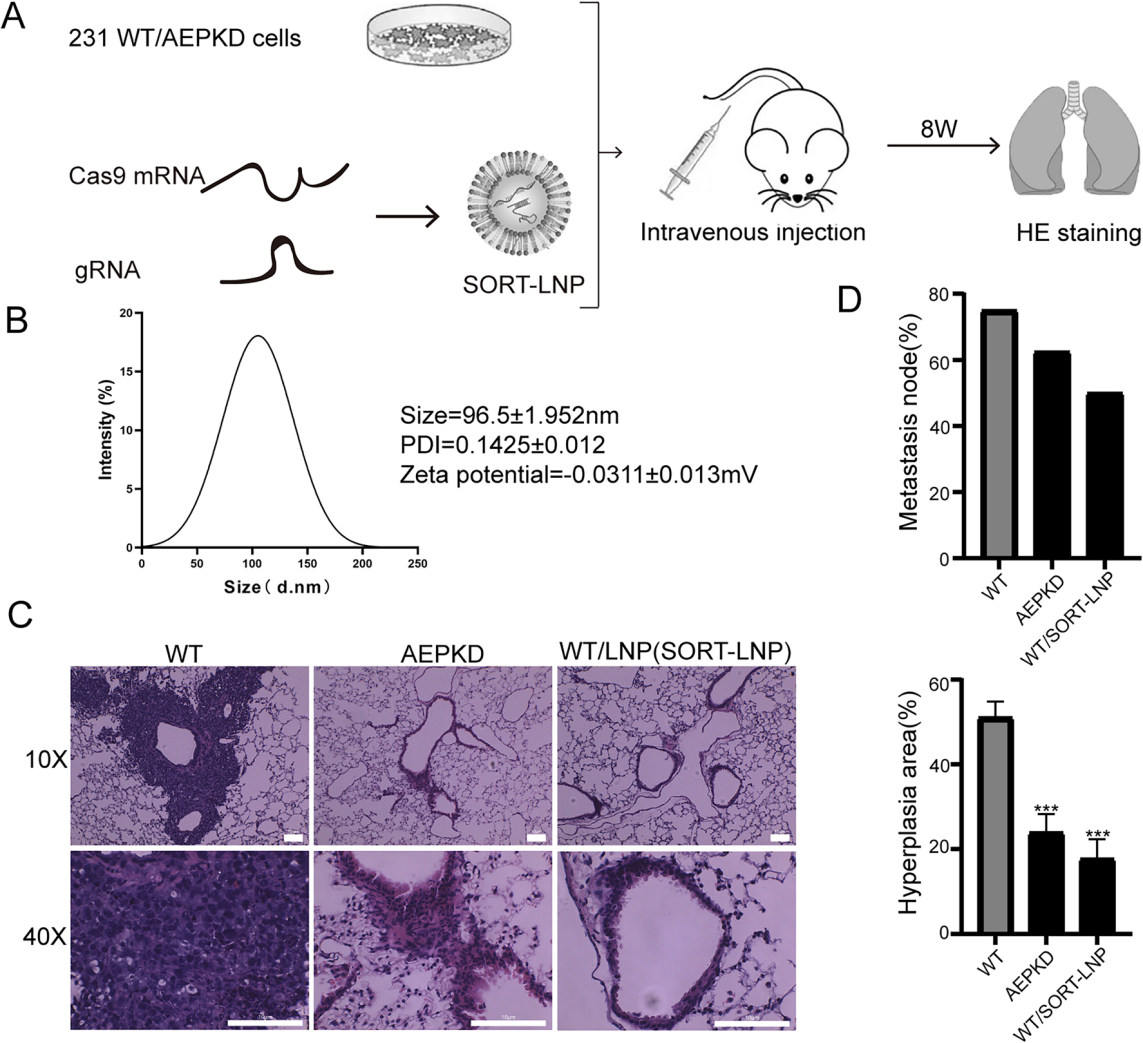
Effect of in-vivo LGMN gene-editing on cancer metastasis by SORT-LNP co-delivered Cas9 mRNA and gRNAs. (**A**) Scheme of experimental work flow. MDA-MB-231 cells were injected into the tail vein of nude mice with or without SORT LNPs targeted to lungs at a dose of 1 mg kg^−1^ total RNA (Cas9 mRNA:gRNAs = 1:2 mass ratio in one formulation) (n = 5). For in-vivo gene-editing of LGMN, SORT LNPs injection was repeated twice at an interval of 2 weeks. AEPKD MDA-MB-231 cells were injected and used as control. (**B**) The particle size, PDI, and zeta potential of SORT LNPs were analyzed by DLS. Data are representative of three independent experiments. (**C**) Representative images of H&E stained lung sections showing micro-metastases/hyperplasia in lungs of mice injected with WT MDA-MB-231 cells, AEPKD MDA-MB-231 cells or WT MDA-MB-231 cells with SORT LNPs respectively. Scale bar: 10 μm. (**D**) Quantification of average metastasis node or hyperplasia area of each group of mice (n = 5). Graphs shown are from one experiment representative of three. Compared with the wild-type group. The data are presented as mean ± SEM. Statistics are unpaired t-test. **P < 0.005, ***P < 0.001, ****P < 0.0001.

The effect of in-vivo Cas9 mRNA/gRNA-mediated LGMN gene-editing on migration and invasion of cancer cells was investigated by experimental lung metastasis assay. To target LNPs to the lung, SORT LNP was used^37^. MDA-MB-231 cells were injected into the tail vein of nude mice, followed with tail vein injection of SORT LNPs targeted to the lungs at a dose of 1 mg kg^−1^ total RNA (Cas9 mRNA:sgRNA = 1:2 mass ratio in one formulation) (Fig. 5A). SORT LNPs injection was repeated twice at an interval of 2 weeks. The particle size, polydispersity index (PDI), and zeta potential of SORT LNPs as assessed from DLS was about 96.5 ± 1.952 nm, 0.142 ± 0.012 and − 0.0311 ± 0.013 mV, respectively (Fig. 5B). As controls, mice were injected via tail vein with MDA-MB-231 WT cells or MDA-MB-231 AEPKD cells (LGMN gene-edited in-vitro). Eight weeks after injection, the lungs were removed, tissue sections were made, and lung metastases were examined by hematoxylin and eosin (H&E) staining. H&E stained tissue sections showed that WT cells formed unequivocal micro-metastases around blood vessels, where cancer cell extravasation took place (Fig. 5C). In contrast, WT cells in the presence of SORT LNPs, as well as AEPKD cells, only led to slight hyperplasia around blood vessels (Fig. 5C). The metastasis/hyperplasia area in the lungs of mice injected with WT cells was significantly larger than that in the lungs of mice injected with WT cells in the presence of SORT LNPs or AEPKD cells (Fig. 5D). The results show that the invasion and migration of MDA-MB-231 cells were significantly reduced by in-vivo Cas9 mRNA/gRNAs mediated LGMN gene editing.

## Discussion

A large part of the failure in breast cancer treatment is due to the metastatic spread of cancer cells, making it crucial to inhibit their metastasis. AEP is overexpressed on the cell surface and in cytoplasmic vesicles in various solid tumors, including breast cancer^38^, and is associated with increased invasiveness and aggressive behavior in several cancers^12^.

In this study, we designed gRNAs specific to legumain (or AEP). Two templates were designed for in-vitro transcription (IVT) of gRNA: linearized pUC57-T7-gRNA and T7-gRNA oligos. The effectiveness of gRNA was verified in various ways to enhance gene editing efficiency.

gRNAs derived from the latter were more effective, probably due to it produced uniform gRNAs after IVT. Cas9 plasmid was modified and optimized. These modifications can enhance the translation efficiency and stability of mRNA molecules in cells^39,40^, as well as improve the quality and yield of mRNA. In addition, co-delivery of Cas9 mRNA and guide RNA (gRNA) by lipid nanoparticles (LNP) to increase the efficiency and stability of RNA delivery^41^, making in-vivo gene editing more efficient than AEPKD cells. Compared to plasmids, Cas9 mRNA and guide RNA are more efficient for editing the LGMN gene to knockout Legumain (or AEP) protein expression in breast cancer cells^42^. They can be directly translated into proteins in the cytoplasm without entering the nucleus^42^. Legumain protein is localized mainly to the endo-lysosomal system^43^, studies have demonstrated that Legumain is initially synthesized as an inactive zymogen and subsequently undergoes proteolytic activation through autocleavage upon reaching the acidic environment of the lysosome^44^. So the pH environment is very important for AEP activation^29,45,46^. We observed that serum starvation reduced AEP activation in cells, likely due to an increase in intracellular pH^47^, which in turn weakens AEP activation, which is a topic that needs further exploration. In-vitro verification was conducted to confirm the impact of AEP knockout on lysosomal and autophagic degradation in tumor cells. Impaired lysosomal function can weaken autophagy and cause the accumulation of various damaged substances^48^, leading to a range of diseases^49–51^. Moreover, electron microscopy experiments further confirmed that the number of autophagic vesicles increased and the degradation of their contents decreased under conditions of autophagy induction. The migration, invasion, and proliferation abilities of tumor cells with AEP knockout were evaluated. Finally, the migration and invasion capacity of cancer cells in-vivo was examined after Cas9 mRNA/gRNA-mediated LGMN gene editing through a lung metastasis experiment. These findings indicate in-vivo CRISPR/Cas9 gene editing technology can inhibit tumor metastasis, providing new ideas and methods for treating tumors. But the specific mechanism remains unclear. Previously, it has been reported that AEP ubiquitination by TRAF6 facilitates its secretion which in turn facilitates tumor invasion and metastasis through degrading extracellular matrix^9^. Moreover, AEP is localized at the apex of invading cells, forming a complex with integrins expressed on lamellipodia and invadopodia^11^, both enhances cancer cell invasion and metastasis.

CRISPR/Cas9 gene editing technology will be widely used in treating human diseases in the future. But the technology is currently highly controversial^35^, the immaturity of gene editing technology will cause many problems such as off-target effect^52^ and organ toxicity^53^. At the same time, unreasonable application will also bring some irreversible effects to human beings. Therefore, through continuous technical improvement, it is hoped that these problems can be effectively solved. We should properly apply this technology in the field of cancer gene therapy.

## Methods and materials

### Cell culture

HEK293T cells, 231-MDA-MB cells Hela cells were purchased from ATCC and were cultured in DMEM(Gibco). The medium was supplemented with 10% fetal bovine serum (Gibco), 2 mM l-glutamine (Gibco), 100 U/ml penicillin and 100 μg/ml streptomycin (Gibco). All cells were culture at 37 °C under 5% (v/v) CO_2_.

### Designing gRNA

The LGMN gene sequence was downloaded from the NCBI gene database and target sequence, usually exon 1, was selected. The gRNA sequence located 20 bases upstream of the PAM (5′-NGG-3′). A pair of gRNAs was used for gene editing instead of single gRNA. The optimal gRNA pair sequences are listed below: sgRNA1: 5′-ATGTTTATCAGTTCTTGTTG-3′, sgRNA2: 3′-AGAAGTCCTAGTAGATATCC-5′. gRNA oligos were synthesized by the Sangon Biotech company (Shanghai, China).

### PGL3-2U6-gRNA plasmid construction

A 434 bp targeted fragment containing a pair of gRNAs, sgRNA1-scaffold-U6 promoter-sgRNA2, was amplified from the pUC57-U6 plasmid (Addgene #115520) using primers containing the gRNA sequences. The primers were designed as follows: F 5′-ATGCGTCTCGAAACATGTTTATCAGTTCTTGTTGCGGTGTTTCGTCCTTTCCACAAG-3′ and R 3′-ATGCGTCTCAACCGCCTATAGATGATCCTGAAGAGTTTTAGAGCTAGAAATAGCAAG-5′. PCR amplification was performed using Phanta Max Super-Fidelity DNA polymerase (Vazyme, China). PCR conditions consisted of an initial denaturation step at 95 °C for 45 s, annealing step at 55 °C for 15 s, 45 s at 72 °C, 32 cycles of 15 s at 95 °C, 55 °C for 15 s and 45 s at 72 °C, and a final elongation step of 5 min at 72 °C. The amplified sgRNA1-scaffold-U6 promoter-sgRNA2 sequence was then inserted between the U6 promoter and the gRNA scaffold in the backbone plasmid pGL3-U6 (Addgene #51133) by restriction enzyme (NEB) cloning to obtain pGL3-2U6-gRNA.

### Construction of templates and IVT of gRNA

Two templates were designed for in-vitro transcription (IVT) of gRNA: linearized pUC57-T7-gRNA and T7-gRNA oligos.

Construction of pUC57-T7-gRNA template: The complementary oligonucleotides with IIS restriction enzyme sites were annealed in 10× Reaction buffer by heating to 95 °C for 5 min, followed by a 2 °C/s ramp down to 85 °C and a 0.1 °C/s ramp down to 25 °C. and the resulting annealed oligonucleotides were inserted into plasmid pUC57 between T7 RNA polymerase promoter and gRNA scaffold by T4 DNA ligase (NEB), 37 °C for 2 h. The gRNA expression plasmid was linearized using DraI restriction enzyme (NEB), 37 °C for 4 h. The linearized plasmid was used as a DNA template for IVT of the gRNA, ensuring that everything is RNase-free.

Construct the T7-gRNA oligos template: A 60-base oligonucleotide is designed for gRNA, which includes the T7 promoter, a 20-base gRNA, and an overlapping region that matches the constant oligonucleotide. In addition, the design of an 80-base constant oligonucleotide sequence containing gRNA scaffold is required for gRNA. We used the Phanta Max Super-Fidelity DNA Polymerase (Vazyme) and primers to amplify the T7-gRNA-gRNA scaffold using annealed oligos as template by PCR. The primers were designed as follows: F 5′-TAATACGACTCACTATAG-3′ and R 5′-AAAAGCACCGACTCGGTGC-3′. PCR conditions consisted of an initial denaturation step at 98 °C for 40 s, annealing step at 51 °C for 10 s, 10 s at 72 °C, 30 cycles of 10 s at 98 °C, 51 °C for 10 s and 10 s at 72 °C, and a final elongation step of 2 min at 72 °C. The PCR products were then used as template for IVT, ensuring that everything was RNase-free. All oligonucleotide sequences and primers were synthesized by the Sangon Biotech company (Shanghai, China).

IVT of gRNA was performed using Hyperscribe™ High Yield RNA Synthesis Kit according to the manufacturer's instructions (APExBIO). After IVT, gRNA was purified by ammonium acetate precipitation (50 mM) and stored at − 80 °C until use.

### Cas9 plasmid modification, IVT and protein expression verification

pST1374-N-NLS-flag-link-Cas9(Addgene, #44758) was used as the starting material, which contained a bacterial promoter T7 promoter located between the eukaryotic promoter CMV and the start condon. The Cas9 plasmid was modified by adding a 3′UTR and a polyA tail to the end of the open-reading-frame of pST1374-N-NLS-flag-linker-Cas9 using seamlessly cloning Kit (Beyotime). To facilitate co-transcriptional capping of mRNA, a point mutation was introduced to change the trinucleotide GGG following the T7 promoter in the plasmid to AGG, so that the co-transcriptional capping reagent EZ Cap™ Reagent AG (APExBio) could be used. The primers were designed as follows: F 5′-ATGCGTCTCCGGGAAATAAGAGAGAAAAGAAGAG-3′ and R 5′-ATGCGTCTCCTCCCTATAGTGAGTCGTATTAATTTCG-3′. Condition of reaction consisted of 94 °C for 90 s, 55 °C for 30 s, and a final step of 15 min at 68 °C. After that, we mutated the trinucleotide GGC to AAC by point mutation, the primers were designed as follows: F 5′-AGGGAGACCCAAGCTAACTAGCACCATGGACAAG-3′ and R 5′-CTTGTCCATGGTGCTAGTTAGCTTGGGTCTCCCT-3′, and the reaction condition was the same as above. The modified Cas9 plasmid was linearized using the AgeI restriction enzyme (NEB) and used as a template for in-vitro transcription (IVT) of Cas9 mRNA. The Cas9 mRNA was made via IVT using T7 RNA polymerase (APExBIO). After IVT, the mRNA was purified by ammonium acetate (50 mM) precipitation.

Cas9 mRNA transfection was carried out according to the instructions of the Lipofectamine 3000 transfection reagent (Invitrogen, USA). The expression of transfected Cas9 mRNA was analyzed by western blotting. The extracted protein was incubated with specific primary antibodies to spCas9 (GenScript, A01935-40, 1:500) and tubulin (ORIGENE, F001, 1:3000). Secondary antibody HRP-Donkey-anti-mouse was used. The Proteins were visualized with an ECL substrate using Tanon imaging system.

### Validation of gene editing

231-MDA-MB and Hela cells were seeded in 6-wells plates at a density of 1 × 10^5^ cells per well about 4–5 h before transfection. Plasmid and mRNA transfection was carried out according to the instructions of the Lipofectamine 3000 transfection reagent (Invitrogen, USA). The dual plasmids encoding gRNAs and Cas9 were co-transfected into breast cancer cell line MDA-MB-231 for about 4–6 h, the medium was replaced with antibiotic-free medium. The cells were collected 36 h after gene editing. gRNAs and Cas9 mRNA co-transfection were performed as above. Genomic DNA was extracted from the cells and the target sequence was amplified by PCR, using the Phanta Max Super-Fidelity DNA polymerase (Vazyme, China). The primers were designed as follows: F 5′-CAGGTGGATGTGCAGCATTG-3′ and R 5′-TGGCAGGAGGTTCCAGAATG-3′. The amplified product was analyzed by 2% agarose gel electrophoresis, and the DNA bands were visualized using a chemiluminescence imaging system (Tanon).

### Western blotting analysis

Gene-edited cells were seeded in 6 cm dishes at a density of 5 × 10^5^ cells per dish for 24 h. The cells were lysed in cell lysis buffer (150 mM NaCl, 12.5 mM β-glycerophosphate 20 mM Hepes pH7.4, 1.5 mM MgCl_2_, 2 mM EGTA) supplemented with protease inhibitor (APExBIO). Cell lysates were centrifuged at 12,000×*g*, 4 °C for 20 min, and the resulting supernatant was collected. The protein concentration in the supernatant was measured with a bicinchoninic acid (BCA) protein assay kit (Thermo Fisher Scientific). The proteins were loaded and separated using 12% SDS–polyacrylamide gel and transferred to a polyvinylidene difluoride membrane (Sigma–Aldrich). The membranes were blocked by 5% fat-free milk dissolved in TBS. The membranes were incubated overnight at 4 °C with primary antibodies against hAEP (R&D Systems, AF2199, 1:2000), LC3 (MBL, DM036, 1:1000), LAMP2 (HUABIO, HN1228, 1:2000), GAPDH (Proteintech, 21000453, 1:100000). After that, HRP-Donkey-anti-goat (Jackson, 146666, 1:100000), HRP-Goat-anti-rabbit (Jackson, 146887, 1:100000) and HRP-Donkey-anti-mouse (Jackson, 146022, 1:100000) were incubated. Then, the Proteins were visualized with an ECL substrate using Tanon imaging system. and the results were analyzed with imageJ software.

### Enzymatic activity assay

Gene-edited cells were washed three times with 1× PBS before lysis, cell lysates were diluted in 0.2 M Na citrate buffer pH 4.0 containing 1% Trion X-100. To measure AEP activities 10 μg of total protein were incubated in 100 μl assay buffer (0.2 M Na citrate buffer pH 4.0, 1 mM DTT) containing 10 μM AEP substrate [Z-Ala-Ala-Asn-7-amino-4-methylcoumarin(AMC)]. Enzymatic activity was measured using microplate reader (TECAN Spark). The excitation wavelength is 380 nm and the emission wavelength is 528 nm.

### T7E1 Cleavage Assay

The genomic DNA were amplified using extracted genomic DNA as template. The PCR products were annealed in 10× Reaction buffer by heating to 95 °C for 5 min, followed by a 2 °C/s ramp down to 85 °C and a 0.1 °C/s ramp down to 25 °C. The annealed samples were digested by T7 Endonuclease I (Beyotime) at 37 °C for 30 min, followed by incubating at 85 °C for 15 min to stop the reaction.

### Immunofluorescence staining and confocal microscopy

Gene-edited cells (5 × 10^4^ cell/coverslip) were fixed with 4% paraformaldehyde in PBS at room temperature for 20 min. The cells were washed three times with PBS. Cells were permeabilized with Digitonin (Sigma) in PBS for 20 min at room temperature, washed three times with PBS and then incubated for 20 min at room temperature in blocking buffer (1.5% BSA in TBS). The cells were stained overnight at 4 °C using a primary antibody against LAMP2 (Santa Cruz Biotechnology, SC-18822, 1:300). The coverslips were washed three times with PBS and then stained with the Alexa-488-conjugated secondary antibody(Invitrogen, 52697A, 1:300), which contained DAPI (Sigma, 1:500), foil wrapped to protect from light, for 3 h at room temperature. Coverslips were washed three times with PBS, mounted on glass slide. The cells were observed using 63-fold Oil objective on Leica Confocal microscope (TCS SP8, Germany). The integrated density is calculated by ImageJ software, which is the mean fluorescence intensity.

### Lysotracker labeling assay

Gene-edited cells were seeded in 24 well plates at a density of 5 × 10^5^ cells per well for 24 h. Lysotracker (Beyotime, C1046) was diluted in DMEM medium at a ratio of 1:1000. After staining for 10 min, the Hoechst nuclear stain (Thermo) was added to the medium at a ratio of 1:500. After incubating for 10 min at 37 °C, the cells were fixed with 4% paraformaldehyde in PBS at room temperature for 20 min. The fluorescence intensity was observed using a confocal microscope, and representative cells were selected and photographed.

### Electron microscope assay

Gene-edited cells were seeded in a 6 cm dish and grown overnight at 37 °C. The cells were then treated with DMEM for 16 h. Cells were fixed with 2.5% glutaraldehyde for 5 min, followed by scraping off the adherent cells and centrifugation to remove supernatant. The cells were then fixed with 2.5% glutaraldehyde at room temperature for 30 min away from light.

### Colony-formation assay

Gene-edited cells were seeded in 6-well plates (100 cells/well, 200 cells/well). After incubation at 37 °C 5% CO_2_ for 2 weeks, the cells were briefly washed with PBS and fixed with methanol at room temperature for 30 min. Cells were then were stained with 0.5% crystal violet at room temperature for 20 min. Finally, the colonies on the plates were scanned and counted.

### Wound healing assay

Gene-edited cells were seeded in 6-well plates. When the cells reached 90–100% confluence, a scratch was made using a 200 μl sterile pipette tip in the center of each well. The cells were washed three times with 1× PBS (0.1 M PBS, pH 7.4) to remove cellular debris and fresh serum-free medium was added. Images were acquired using a microscope (NIB610-FL, Nexcope) at 0, 6, 12, and 24 h of culture. Cell migration was determined by calculating the percentage of wound closure in three independent experiments using ImageJ software.

### Transwell assay

Transwell assay was conducted as follows: 10% FBS medium was added to the lower chamber, and gene-edited cells (5 × 10^4^ /well) in serum-free medium were added to the upper chamber. After incubation at 37 °C with 5% CO_2_ for 24 h, the cells on the lower surface were fixed with 4% paraformaldehyde and stained with 0.5% crystal violet for 20 min. The transwell was washed three times with PBS and imaged using a microscope (NIB610-FL, Nexcope).

### Nanoparticle formation and stability testing

LNP/RNA preparations were formulated using 50% DOTAP SORT LNPs, which targeted the lungs. Specific formulations were prepared as described previously^37^. To study LNP stability, the zeta potential, size distribution, polydispersity index were monitored. 50% DOTAP SORT LNPs were dialyzed with 1× PBS and then diluted to 1 ng/μl mRNA (Cas9 mRNA: gRNA = 1:2). Then, 1 ml was pipetted into DLS ultramicro cuvettes for monitoring zeta potential, particle size, and polydispersity index.

### In-vivo lung metastasis assay

Female nude mice weighing 18–20 g were randomly divided into three groups: n = 6–8 per group. The mice were maintained at the Laboratory Animal Research Center, complying with the animal guidance and regulations of Dali University. Two groups were injected with 1 × 10^6^ MDA-MB-231 WT or MDA-MB-231 AEPKD cells each mouse via tail vein. The last group was injected with SOTR-LNP (lung) following MDA-MB-231 WT cells injection at a dose of 1 mg kg^−1^ total RNA (Cas9 mRNA:sgRNA = 1:2 mass ratio) in one formulation. SORT LNPs injection was repeated twice at an interval of 2 weeks. After 8 weeks, mice were anesthetized, perfused with 4% paraformaldehyde. The lungs were removed for paraffin section and HE staining.

### Ethical statement

All animal experiments in this study were approved by the Ethics Committee of Dali University. The animal experiments complied with the ARRIVE guidelines and were carried out in accordance with EU Directive 2010/63/EU for animal experiments.

### Statistical analysis

GraphPad Prism 8 was used for statistical analysis and plotting graphs, Quantitative image analysis of tissue distribution was conducted using ImageJ software. Analysis of differences was carried out using Student’s t-test or one-way analysis of variance (ANOVA). The difference was referred significance *, **, ***, **** if P < 0.05, P < 0.005, P < 0.001, P < 0.0001, respectively.

### Supplementary Information

Supplementary Figure 1.

## Data Availability

Data are available upon request. Source data may be obtained from the corresponding author on reasonable request.
