# Supplementary material for: Co-delivery of Cas9 mRNA and guide RNAs for editing of LGMN gene represses breast cancer cell metastasis
Source: Sci Rep. 2024 Apr 6;14:8095. doi: 10.1038/s41598-024-58765-6 (PMC10998893; doi:10.1038/s41598-024-58765-6)

The whole uncropped images of the original western blots

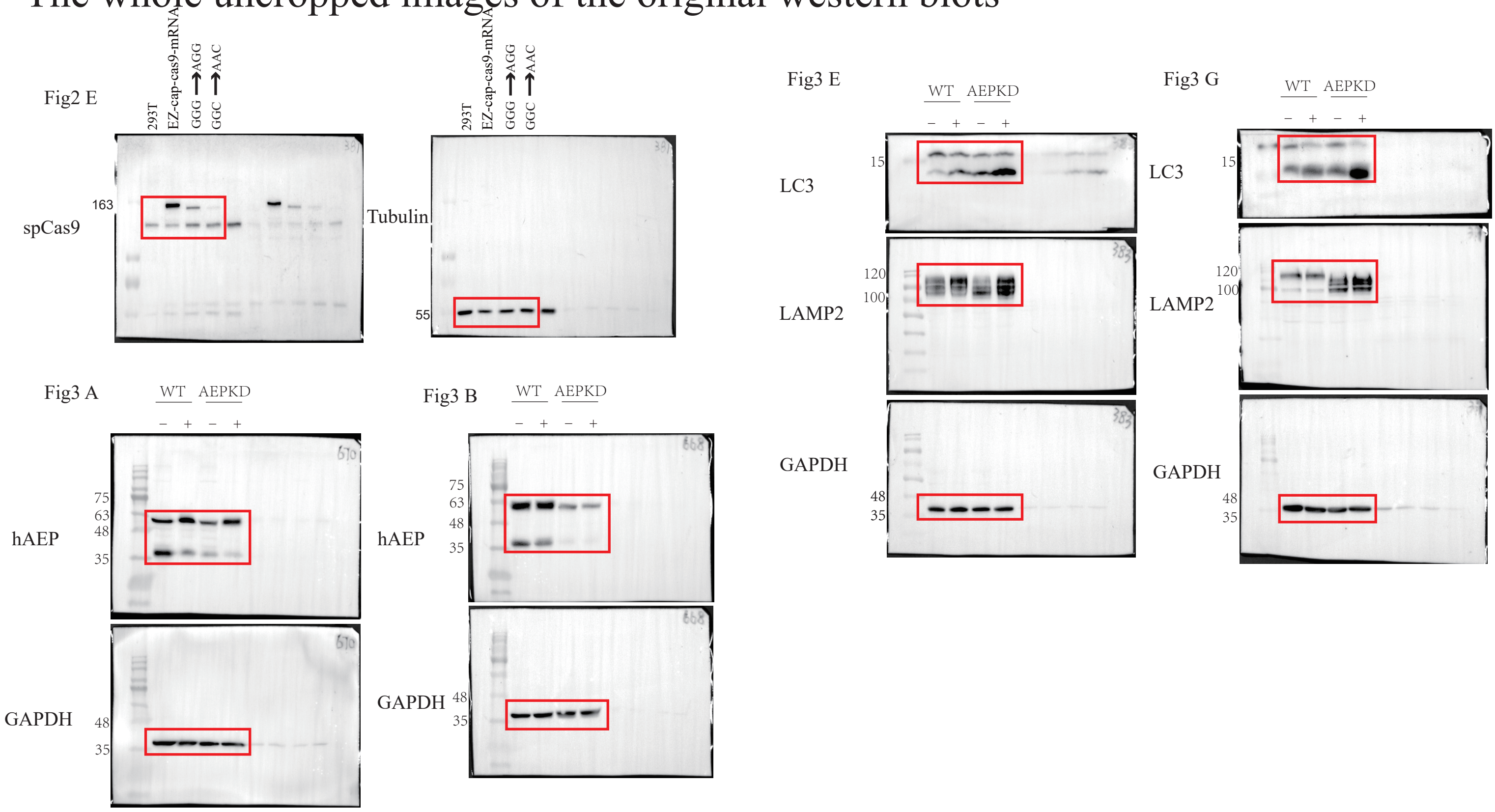

The whole uncropped images of the original Electrophoretic gels

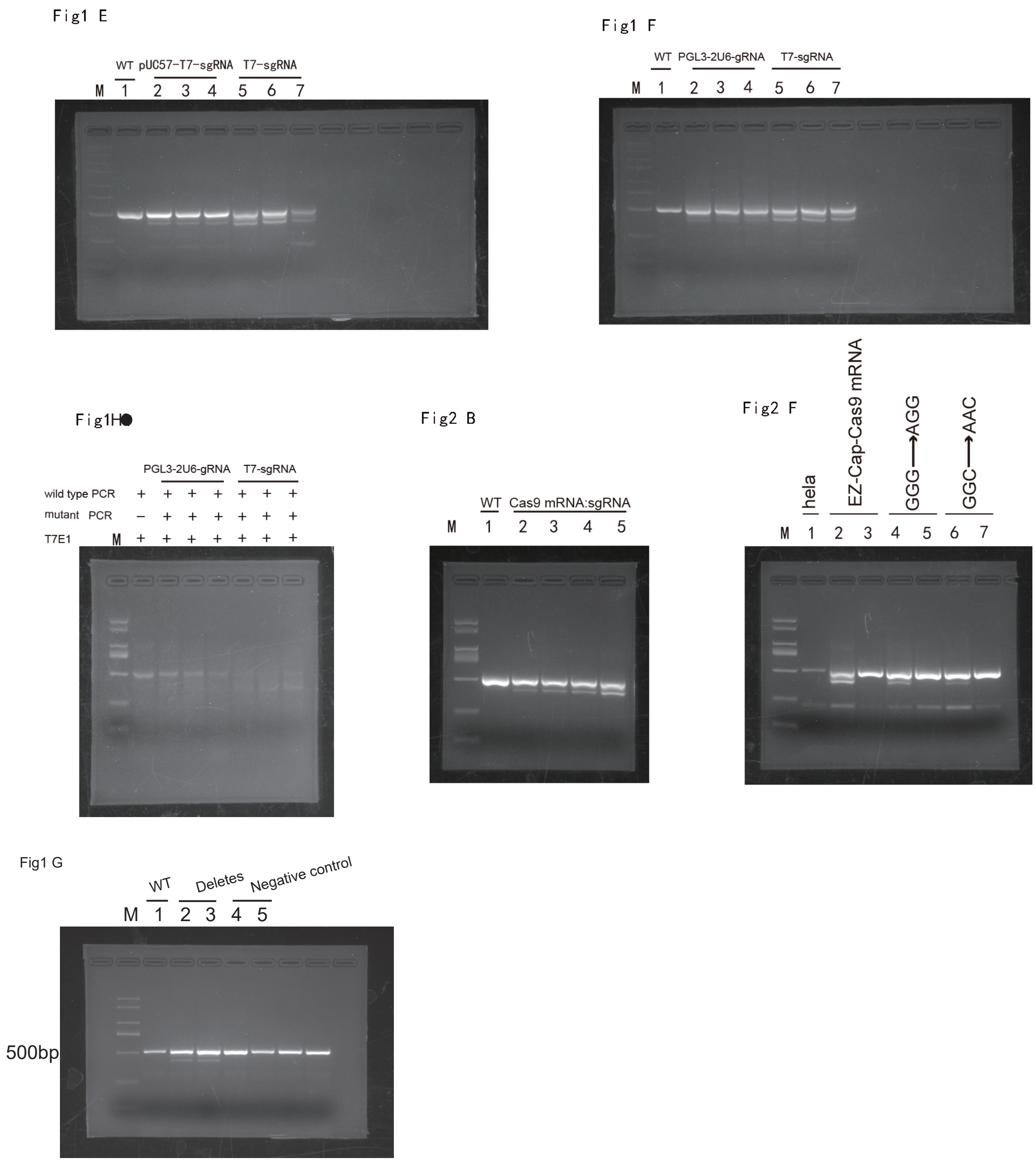

Supplement: Supplementary file 1 — Supplementary Figure 1. [file 41598_2024_58765_MOESM1_ESM.pdf]
